# A New Perspective on the Role of *Lactobacillus acidophilus* in the Prevention and Treatment of Allergic Diseases and Cancer

**DOI:** 10.3390/biom16070930

**Published:** 2026-06-23

**Authors:** Remigiusz Olędzki, Kristi Kerner

**Affiliations:** 1 Department of Biotechnology and Food Analysis, Wroclaw University of Economics and Business, Komandorska 118/120, 53-345 Wroclaw, Poland; 2 Chair of Food Science and Technology, Institute of Veterinary Medicine and Animal Sciences, Estonian University of Life Sciences, Fr. R. Kreutzwaldi 56/5, 51006 Tartu, Estonia

**Keywords:** *Lactobacillus acidophilus*, cancer, allergies, exopolysaccharides, macrophages, dendritic cells, lymphocytes T, immunotherapy

## Abstract

The aim of this review is to provide a narrative analysis of the role of *Lactobacillus acidophilus* as an active modulating factor in the prevention and treatment of cancer and allergic diseases. The paper discusses the molecular, metabolic, and bionanotechnological mechanisms of *Lactobacillus acidophilus*’s anticancer and immunomodulatory effects, which define this probiotic as an essential component of modern natural and functional medicine. A narrative review of the scientific literature was conducted, mainly from 2019–2026, focusing on the results of *in vitro* studies and studies on preclinical *in vivo* models, which analyzed the effect of live *L. acidophilus* strains, tyndallized bacteria (paraprobiotics) and cell-free supernatant from *L. acidophilus* cultures on, among others, immune system signaling pathways, tissue cytokine profile, and the integrity of the gastrointestinal epithelial cell barrier (enterocytes). Results indicate that *L. acidophilus* exerts significant antiallergic, antiproliferative, and proapoptotic effects against many types of cancer. Among other aspects, the ability of *L. acidophilus* to stimulate the production of anticancer exopolysaccharides and short-chain fatty acids, which directly influence the functioning of immune cells, is covered. The article thoroughly explains the immunomodulatory effects of *L. acidophilus* and the ability of this probiotic to regulate cytokine profiles, which helps promote an anti-inflammatory environment crucial for maintaining intestinal homeostasis. The article also discusses the direct interaction of *L. acidophilus* with immune cells, such as dendritic cells and macrophages, which leads to their activation and subsequent influence on the differentiation of T lymphocytes, which play a key role in the regulation of immune processes and in the development of immune tolerance. *L. acidophilus* is a universal mediator of immunological and metabolic homeostasis. Its ability to synergize with conventional therapies (chemotherapy, oncolytic virotherapy) and its innovative applications in the creation of postbiotics and paraprobiotics may provide a new approach to the treatment of inflammatory, allergic, and neoplastic diseases. Further clinical studies are necessary to assess the efficacy, safety, and optimal dose of this probiotic, which are essential for the widespread use of *L. acidophilus* in human therapy.

## 1. Introduction

The human body functions in close symbiosis with the microorganisms that inhabit it. Of particular importance in this symbiosis is the gut microbiota, a population of approximately 10^14^ microorganisms, which is approximately 10 times greater than the total number of human cells in the body [1].

Recent research clearly indicates that proper intestinal bacterial flora is crucial not only for proper digestion, but also for the course of immune processes (including the prevention of diseases, such as autoimmune diseases) [2], maintaining well-being, and even proper functioning and regeneration of the brain (e.g., after an ischemic stroke) [3].

It has been confirmed that gut microbiota participates in the fermentation of dietary fiber (which is not digested and absorbed in the small intestine), leading to the formation of short-chain fatty acids (SCFAs), the main source of energy for intestinal epithelial cells and a key factor in maintaining proper intestinal function [4].

Additionally, the correct composition of human intestinal flora inhibits inflammation in the intestines and constitutes a physical and biochemical barrier protecting the intestinal mucosa and the organs surrounding the intestines from harmful microorganisms. It has been proven that intestinal microbiota influences the development and maturation of immune system cells, stimulating antibody production, and regulating inflammatory responses. On the other hand, dysbiosis of the intestinal microbiota caused by, among others, aging of the body, unfavorable BMI (body mass index), the use of a diet based on highly processed food and the abuse of drugs or stimulants is a confirmed factor contributing to the occurrence of inflammatory diseases [5].

It has been confirmed that 70 to 80% of immune system cells reside directly in the intestines, in organized tissues such as Peyer’s patches (clusters of lymphoid follicles) in the ileum and the lamina propria of the small intestinal mucosa. Studies have shown that a lack of microbiota diversity in childhood significantly increases the risk of allergies, asthma, and autoimmune diseases later in life [6].

The human gut is often called the “second brain.” This stems from the presence of a specific bidirectional signaling pathway between the gut and the central nervous system, referred to as the “gut–brain axis” or “microbiota–gut–brain axis.” Gut microbiota can produce large amounts of neurotransmitters, such as serotonin, gamma-aminobutyric acid (GABA), and dopamine, which directly influence mood and behavior, including by increasing or decreasing impulsive behavior (such as aggression) [7].

It has been confirmed that approximately 90% of serotonin, often referred to as the “happiness hormone”, is produced in the intestines by specialized enteroendocrine cells known as enterochromaffin cells (EC cells), which are located in the intestinal mucosa and respond to both ingested nutrients and metabolites produced by the intestinal microbiota [8].

Research shows that individuals with obesity typically have a different gut microbiota composition compared to individuals of a healthy weight. In obese individuals, the gut microbiota becomes dominated by bacteria capable of intensively extracting energy from food, such as *Lactobacillus reuteri*, while the number of *Bifidobacterium animalis*, *Methanobrevibacter smithii*, and *Lactobacillus acidophilus* 5 (LA5) decreases, which can consequently lead to excessive fat accumulation and weight gain [9]. In people suffering from obesity, a significant decrease in the number of bacteria of the genera *Akkermansia*, *Faecalibacterium*, *Oscillibacter*, *Alistipes* and *Bacteroides faecalis* is also observed compared to people with normal body weight [10]. Therefore, it is indicated that differences in the composition of the intestinal microbiota may also be an early diagnostic marker in the treatment of type 2 diabetes in patients at high risk of this disease [11]. At the same time, intensive research is being conducted to answer the question of whether intestinal microbiota transplantation from healthy individuals (with correct body weight) can be a potential method for treating obesity [12].

*Lactobacillus acidophilus* is a species of probiotic bacteria that is currently undergoing intensive research to explain the mechanisms of modulation of the immune balance (which is crucial in allergic diseases) and the mechanisms of strengthening the function of the immune system towards the identification and elimination of cancer cells.

The aim of this article is to summarize and critically evaluate the latest scientific reports on the mechanisms by which *L. acidophilus* influences the immunity of the human body. The article also provides current knowledge on the effects of *L. acidophilus* and scientifically proven recommendations for the use of *Lactobacillus* probiotics.

## 2. Materials and Methods

Allergic conditions most often result from an overactive immune response, primarily involving Th2 lymphocytes, which are part of the humoral immune response [13]. Cancer, on the other hand, is the result of a weakened immune response, including a decrease in Th1 (T-helper 1) cellular immunity and a decrease in the activity of cytotoxic NK (Natural Killer) lymphocytes. In this article, we want to demonstrate that *L. acidophilus* can exert immunomodulatory and bidirectional effects, both alleviating allergies and stimulating the body to fight cancer [14]. To prepare this article, a narrative literature review was conducted using four electronic databases: Scopus, PubMed, Web of Science and Google Scholar. The analysis used articles on both preclinical studies and randomized controlled clinical trials that investigated the effects of different *L. acidophilus* strains in the prevention and treatment of allergic diseases and cancer. The literature analysis included publications primarily from 2019–2026. The following Boolean combinations of terms were used: *Lactobacillus acidophilus* AND allergy, *Lactobacillus acidophilus* AND allergies, *Lactobacillus acidophilus* AND allergic AND diseases, *Lactobacillus acidophilus* AND cancer, *Lactobacillus acidophilus* AND cancer AND colon, *Lactobacillus acidophilus* AND cancer AND bowel, *Lactobacillus acidophilus* AND cancer AND liver, *Lactobacillus acidophilus* AND cancer AND cervical, *Lactobacillus acidophilus* AND cancer AND breast. For all given Boolean combinations, versions were used in which the phrase “*Lactobacillus acidophilus*” was replaced with the word “probiotic.” A manual literature search using single thematic keywords was also used in the study to prevent the exclusion of articles that used synonyms for the previously mentioned main keywords. Only full text articles were used to prepare the manuscript.

## 3. Mechanisms of the Modulation of the Immune Response Induced by *Lactobacillus acidophilus*

The probiotic bacteria *L. acidophilus* can influence the immune system both directly, through interactions with the intestinal wall, and indirectly, through the metabolites they produce. It has been observed that modulation of the immune response by *L. acidophilus* may occur through the influence on selected cells of the immune system, such as dendritic cells, macrophages, natural killer cells, γδ T cells, B cells and Tregs or Th17 cells [15]. *L. acidophilus* may also exert a protective effect on the body not only by regulating the cells of the innate and adaptive immune system, but also by limiting intestinal permeability and dysbiosis, maintaining immune system homeostasis [16].

### 3.1. Activation of Antigen-Presenting Cells (APCs) and Cytokine Modulation

The role of professional antigen-presenting cells (pAPCs) is to integrate innate and adaptive immune responses through phagocytosis of pathogens and the digestion of their lipopolysaccharides into smaller fragments and proteins into peptides [17]. The presentation of these antigens in complex with specialized glycoprotein MHC class II (Major Histocompatibility Complex) molecules is a key element in T cell activation and the initiation of a specific immune response [18]. It has been confirmed that Lactobacillus acidophilus can both directly influence the production of antigen-presenting cells (APCs), such as dendritic cells, macrophages, and B lymphocytes, and can also stimulate them to secrete specific cytokines, such as interleukin-12 (IL-12), which promotes a strong Th1 cellular immune response (inflammatory and antiviral) [19]. On the other hand, *L. acidophilus* can stimulate APCs to secrete interleukin-10 (IL-10), an anti-inflammatory cytokine that promotes the activity of regulatory T lymphocytes (Treg), while also conditioning high immune tolerance of mucous membranes to harmless antigens (e.g., intestinal bacteria or food components) [20].

### 3.2. The Role of Lactobacillus acidophilus in the Activation of Dendritic Cells (DCs)

Probiotics of the *Lactobacillus* genus may have the ability to suppress excessive pro-inflammatory activation induced by pathogens by influencing the production of cytokines [21]. It has been confirmed that *L. acidophilus* (LA-5) and *Lactobacillus rhamnosus* (LR-32) strains can induce immature dendritic cells (DCs) (exposed to pro-inflammatory lipopolysaccharide from *E. coli*) to attenuate the transcription of genes associated with the inflammatory response and apoptosis, such as CASP1, NLRP3, and RIPK1. Consequently, silencing the CASP1 and NLRP3 inflammasome pathways protects tissues from damage and pyroptosis, which typically accompany increased inflammation. Additionally, reducing the transcription of genes controlling the apoptotic and necroptotic pathways (such as RIPK1) allows for cell survival and the maintenance of tissue barrier integrity in the presence of toxins and allergens. On the other hand, it has been observed that probiotic *Lactobacillus* strains can maintain or even increase the transcription and secretion of some key pro-inflammatory cytokines, such as IL1B, IL6, IL12, and CXCL8, by acting on unstimulated dendritic cells that recognize *L. acidophilus* (LA-5) as a signal stimulating their pro-inflammatory response [21]. This allows immune cells to effectively counteract infectious agents while limiting the risk of tissue damage caused by excessive inflammation (the so-called immunomodulatory effect). It is suggested that modifications of the macromolecules of the cell wall of the *L. acidophilus* bacteria in the form of increased glycosylation may have a significant impact on reducing the amount of pro-inflammatory cytokines produced by immune system cells in the bodies of the studied patients [21].

It has also been confirmed that contact of monocytes derived from peripheral blood mononuclear cells (collected from women with endometriosis) with *L. acidophilus* can reduce the production of pro-inflammatory cytokines, such as IL-1 (interleukin-1) and IL-6 (interleukin-6). This beneficial effect was observed after 48 h, although *L. acidophilus* initially (after 24 h) induced a stimulating effect on the production of pro-inflammatory cytokines, which was likely related to the fact that the body may treat *L. acidophilus* as an antigenic factor (antigen) within the first 24 h. It is suggested that modifications of the macromolecules of the cell wall of *L. acidophilus* bacteria in the form of their increased glycosylation may have a significant impact on reducing the amount of pro-inflammatory cytokines produced by immune system cells in the bodies of the studied patients [22]. This study confirms that after an initial phase of temporary stimulation, *L. acidophilus* subsequently causes a weakening of inflammatory responses in the body.

Animal studies in piglets exposed to lipopolysaccharide derived from the outer membrane of Gram-negative bacteria have demonstrated that dietary *Lactobacillus* modulates the colonic microbiota, resulting in increased intestinal butyrate levels and upregulated expression of the short-chain fatty acid receptor GPR43 in intestinal epithelial cells as well as in immune cells, including macrophages, neutrophils, and dendritic cells. As a result of this phenomenon, there is an increased availability of butyrate for intestinal epithelial cells (enterocytes), which consequently leads to a reduced secretion of pro-inflammatory cytokines IL-1β, IL-6 and TNF-α and a reduced expression of inducible nitric oxide synthase (iNOS), which is a key functional marker of macrophages with a pro-inflammatory M1 phenotype. Nitric oxide synthase (iNOS), a characteristic of M1 macrophages with a pro-inflammatory phenotype, utilizes L-arginine to produce NO (a highly reactive form of nitrogen involved in innate immunity), which eliminates pathogenic bacteria and viruses. Conversely, excessive or chronic release of NO can cause significant damage to host tissues [23].

**Figure 1:**
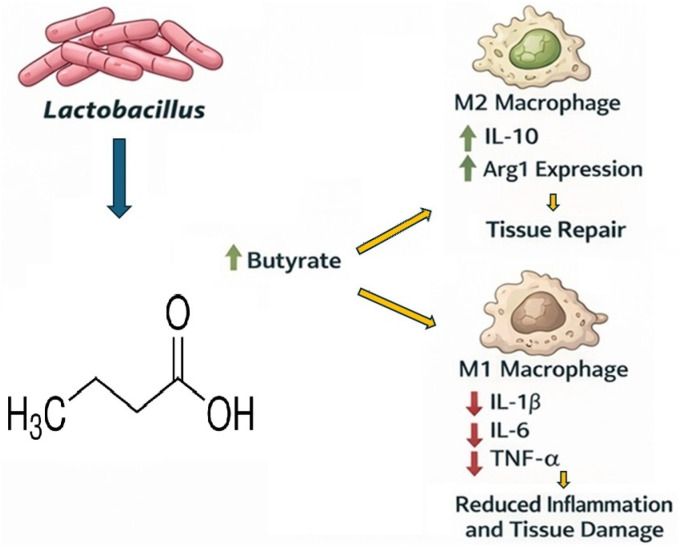
Increased butyrate availability induced by *L. acidophilus* in intestinal epithelial cells modulates cytokine secretion in M1 and M2 macrophages [23].

At the same time, increased butyrate availability leads to increased secretion of the anti-inflammatory cytokine IL-10 and increased expression of the enzyme Arg1 (arginase-1) in M2 macrophages (anti-inflammatory and repair), which uses arginine to produce ornithine, an amino acid essential for collagen synthesis and repair of damaged intestines (Figure 1). It has also been shown that macrophages activated by *Lactobacillus* bacteria (as a result of exposure to LA-derived butyrate) lead to a decrease in the expression of TLR4, MyD88 and phosphorylated NF-κB p65 proteins in intestinal epithelial cells and, at the same time, to an increase in the expression of tight junction proteins, such as ZO-1 and occludin proteins in intestinal epithelial cells [23].

### 3.3. Lactobacillus acidophilus in the Activation of T and B Lymphocytes

It has been confirmed that *L. acidophilus* can participate in the modulation of humoral immunity, which is manifested by the activation of B lymphocytes to produce, among others, secretory immunoglobulin A (sIgA). *L. acidophilus* also participates in the activation of T helper lymphocytes with the CD4+ co-receptor (CD4+ Th lymphocytes), which leads to the production of cytokines that stimulate B lymphocyte proliferation [13].

The results of preclinical studies obtained in animal models (calves and piglets) suggest that multi-strain probiotic mixtures containing *L. acidophilus* can effectively increase the number of B lymphocytes in peripheral lymphatic organs (lymph nodes, spleen, tonsils), in the bone marrow and in peripheral blood, and induce a humoral response in the form of increased IgA and IgG production. However, clinical trials conducted in humans (in men who lead a sedentary lifestyle) did not show significant changes in the number of B lymphocytes in peripheral lymphatic organs (such as lymph nodes, spleen, tonsils), in bone marrow and in peripheral blood after supplementation with a probiotic mixture containing *L. acidophilus* [13]. This observation indicates a clear gap in knowledge and the need for further research into the cellular mechanisms of the human immune response.

**Figure 2:**
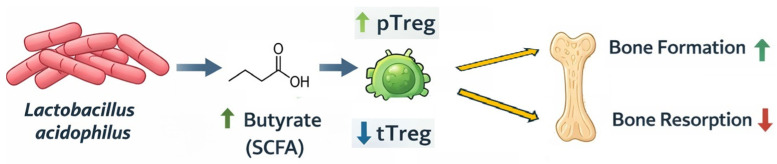
Role of *L. acidophilus* in Bone Homeostasis: Modulation of pTreg and tTreg Balance by butyrate [24].

It has been reported that estrogen deficiency–induced inflammation in women with postmenopausal osteoporosis (PMO) disrupts the balance between thymus-derived regulatory T (tTreg) and peripherally induced regulatory T (pTreg) cells. This imbalance is characterized by a reduction in functional Treg populations and a concomitant increase in Th17 cells, thereby promoting osteoclast-mediated bone resorption. This probably occurs because of the conversion of pTreg lymphocytes called RORγT−pTreg into Th17 cells (Th17 lymphocytes), which have pro-inflammatory and osteoclastogenic effects. Animal model studies (ovariectomized female BALB/c mice aged 8–12 weeks) confirmed that *L. acidophilus* supplementation can significantly restore balance and increase the frequency of pTreg cells along with a reduction in the number of tTreg in the *lamina propria* of the small intestine, large intestine, and within the mesenteric lymph node (MLN) and bone marrow (Figure 2). Furthermore, it has been observed that RORγT−pTreg cells stimulated by the presence of butyrate have reduced osteoclastogenic potential [24].

It has also been confirmed that *L. acidophilus* activates regulatory T lymphocytes, leading to the release of anti-inflammatory IL-10 [25]. In studies using pigs (weaned piglets of the (Landrace × Yorkshire) × Duroc variety, 24–25 days old) that were given pro-inflammatory lipopolysaccharide with food, an increase in the number of CD4+ and CD8+ helper T lymphocytes was observed, which is a consequence of the body’s reaction to the presence of endotoxin. However, simultaneous administration of lipopolysaccharide and *L. acidophilus* to the study animals resulted in a reduction in the total number of leukocytes, primarily CD4+ and CD8+ T helper lymphocytes (compared to animals receiving lipopolysaccharide alone), which may be related to the fact that the analyzed probiotic prevents over-reactivity of the immune system. *L. acidophilus* likely acts by reducing the number of CD4+ and CD8+ T helper cells to a slightly lower and therefore safer level, thereby simultaneously removing the immunogenic substance and counteracting the development of excessive inflammation. Additionally, it was observed that healthy individuals (not exposed to lipopolysaccharides) who took *L. acidophilus* had a slight increase in CD4+ cell count (4 h after ingestion), which may be related to the phenomenon known as “immune priming.” This confirmed that *L. acidophilus* can discreetly stimulate the immune system to produce CD4+ cells, training it to be ready for defensive action, but at the same time not causing unnecessary inflammation [26].

Research indicates that *L. acidophilus* has strong immunomodulatory potential and can attenuate the acute inflammatory response induced by endotoxins through modulation of cytokine production. It has been shown that *L. acidophilus*, by suppressing the activity of the TLR4/NF-κB signaling axis in peripheral blood mononuclear cells (PBMC), can lead to a significant reduction in the production of two key pro-inflammatory mediators, such as TNF-α and IL-6. Of particular relevance to immune homeostasis, probiotic intervention in endotoxin-treated animals modulated the late-phase immune response (12 h after lipopolysaccharide exposure), characterized by decreased expression of pro-inflammatory cytokine genes (IFN-γ, IL-8, IL1B1) and a concomitant increase in anti-inflammatory cytokines (IL-4, IL-10) [26]. This observation confirms that during infection, *L. acidophilus* may exert a dual action, inhibiting inflammatory (pro-inflammatory) signaling while simultaneously enhancing repair and anti-inflammatory mechanisms [10].

It should be noted that excessive and chronic inflammation is highly harmful to the body because, by initiating a cascade of pro-inflammatory cytokines (e.g., TNF-α, IL-6), it can directly stimulate the survival, uncontrolled proliferation, and migration of cancer cells. In allergic conditions, excessive and persistent inflammation can lead to intense tissue infiltration by eosinophils and T lymphocytes, which release cytotoxic substances (such as eosinophil cationic protein, perforins, and granzymes), leading to damage and permanent remodeling of cellular structures (e.g., in the respiratory tract or skin) [10,26].

### 3.4. Lactobacillus acidophilus in Strengthening the Intestinal Barrier

Various strains of *L. acidophilus* have been shown to enhance mucosal homeostasis. This is thought to occur by inducing increased expression of Mucin 2 (Muc2), a glycoprotein that is a major component of intestinal mucus and is secreted by specialized intestinal epithelial cells called goblet cells. Animal studies (eight-week-old male C57BL/6 mice) have confirmed that *L. acidophilus* (in particular the FCQHC4L1 strain) can protect intestinal cells against damage induced by dextran sodium sulfate (DSS), both by promoting the expression and secretion of mucin, but also by reducing the secretion of pro-inflammatory factors, which results in limiting the development of inflammation in the colon. Furthermore, the FCQHC4L1 *L. acidophilus* strain also stimulates the gut microbiota to increase butyric acid secretion, which is the main energy source for intestinal epithelial cells (enterocytes and colonocytes). It has also been shown that conditioned medium containing substances secreted by *L. acidophilus* can inhibit endoplasmic reticulum (ER) stress and ameliorate abnormal MUC2 expression by inhibiting the activation of the GRP78/ATF6 and GRP78/IRE1/XBP1 signaling pathways, which in turn improves intestinal barrier integrity. It was also confirmed that *L. acidophilus* FCQHC4L1 can significantly reduce the level of pro-inflammatory interleukin (IL)-6 and tumor necrosis factor alpha (TNF-α) and increase the level of anti-inflammatory IL-10 in colon tissues affected by inflammatory bowel disease [27].

*L. acidophilus* has been shown to inhibit the formation of defective tight junctions (TJ) in the intestinal epithelium, a major etiological factor of inflammatory bowel disease (IBD). It has been confirmed that *L. acidophilus* may exert protective and therapeutic effects by inhibiting the production of tumor necrosis factor alpha (TNF-α), a key pro-inflammatory cytokine involved in the immunopathology of IBD and which increases the permeability of tight junctions in the intestinal epithelium [28].

Studies using Caco-2 cells (derived from human colon cancer) have shown that the *L. acidophilus* (LA1) strain can prevent the increase in tight junction permeability in intestinal epithelium by inhibiting TNF-α-induced activation of the NF-κB p50/p65 gene in intestinal epithelial cells (enterocytes), as well as by inhibiting the expression of myosin light chain kinase (MLCK), which protects tight junction proteins from translocation and degradation. The *L. acidophilus* (LA1) strain can also prevent the increase in tight junction permeability of the intestinal epithelium by activating the TLR-2 phosphatidylinositol 3-kinase (PI3K) pathway and phosphorylation of IKK-α (i.e., the TLR-2/PI3K/IKK-α signaling axis), which effectively blocks the translocation of the NF-κB factor to the cell nucleus [28]. At the same time, it was confirmed that *L. acidophilus* LA1 can cause TLR-2 and MyD88 protein-dependent activation of NF-κB p50/p65 in immune system cells [28], which confirms that this probiotic is a skillful modulator that can simultaneously stimulate immunity and suppress inflammation in intestinal epithelial cells. The results quoted were obtained from studies conducted on the Caco-2 model (human epithelial cells) and on an animal model (mice), which gives the presented arguments high-quality evidence in terms of the therapeutic role of the *L. acidophilus* LA1 strain.

The results suggest that *L*. *acidophilus* can increase the level of transmembrane glycoprotein MUC-1 in gastric epithelial cells, which is a key component of mucus protecting the gastric epithelium against damage and the impact of pathogens. At the same time, a relationship was confirmed showing that increased production of the protective mucus MUC-1 by the gastric epithelium in response to *L. acidophilus* stimulation is associated with decreased production of galectin-3 (Gal-3), a protein that promotes cancer development [29]. *L. acidophilus* has been shown to reduce galectin-3 (Gal-3) levels. Overexpression of Gal-3 in cells undergoing carcinogenesis promotes tumor progression and metastasis to other tissues and organs [29,30]. Table 1 summarizes the mechanisms of action of *L. acidophilus* and its influence on the modulation of cellular and humoral immunity.

## 4. The Effectiveness of *Lactobacillus acidophilus* in Preventing Allergic Diseases

Specialized strains of the probiotic *L. acidophilus* can be highly effective in treating many types of diseases, such as allergic diseases, inflammatory diseases, autoimmune diseases, cancer, and the aging process.

**Figure 3:**
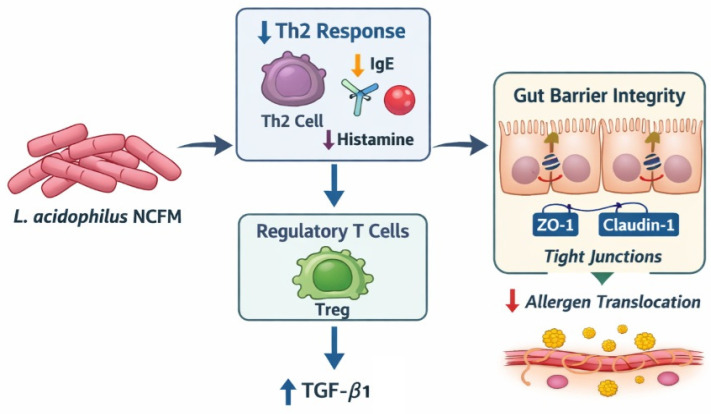
Mechanism of action of the *L. acidophilus* NCFM strain in restoring immune balance in food allergies [31].

It has been confirmed that *L. acidophilus* as a supplement or component of functional foods can support the treatment of food allergies. The NCFM strain of *L. acidophilus* has been shown to be effective against food allergies by affecting both the overactivated immune system and preventing intestinal leakage. It has been observed that the protective effect of the NCFM strain of *L. acidophilus* is based on the mechanism of restoring the immune balance by suppressing the Th2 response (which results in a reduction in the production of IgE and histamine) with simultaneous induction of Treg regulatory lymphocytes and the expression of the anti-inflammatory cytokine TGF-β1 secreted by them (Figure 3). It has also been confirmed that the NCFM strain of *L. acidophilus* strengthens the integrity of the intestinal barrier by upregulating tight junction proteins (ZO-1, claudin-1), which limits the process of allergen translocation (Figure 1). Based on 16S rRNA sequencing analysis, it was shown that the NCFM strain of *L. acidophilus* can also lead to beneficial remodeling of the gut microbiota, leading to an increase in its α-diversity and the promotion of taxa producing short-chain fatty acids (SCFA) [31]. It was confirmed that the phenomenon of gut microbiota remodeling is manifested by an increase in the number of beneficial bacteria such as *Lachnospiraceae* and *Muribaculaceae*, which have the ability to fermentatively degrade dietary fiber and complex carbohydrates and produce immunomodulatory metabolites such as short-chain fatty acids (SCFAs) such as acetate, propionate, and butyrate, which consequently leads to reduced secretion of pro-inflammatory cytokines IL-1β, IL-6, and TNF-α by macrophages with a pro-inflammatory M1 phenotype. Additionally, it was observed that *L. acidophilus* (NCFM strain) contributes to an increase in the number of other SCFA producers, such as *Alistipes*, *Blautia*, and *Lachnospiraceae* [31].

Studies on *L. acidophilus* have shown that the KLDS 1.0738 strain may play an important role in alleviating inflammation induced by β-lactoglobulin, the main whey protein in cow’s milk (approx. 50–55% of whey), which is a strong allergen for the human body. The protective effect of *L. acidophilus* strain KLDS 1.0738 is achieved through the mechanism of increasing the expression of miR-146a, which acts as a negative regulator of the TLR4/NF-kappa B signaling pathway, which leads directly to the inhibition of the production of pro-inflammatory cytokines. For this reason, it is indicated that live cultures of this strain may be an important element of precise therapy for cow’s milk allergy (CMA) resulting from the suppression of food hypersensitivity at the cellular level [32].

It has been shown that *L. acidophilus* can counteract the increased susceptibility to food allergies in the offspring of animals delivered by cesarean section (*sectio caesarea*), which is the result of higher expression of markers such as cytokines IL-4 and IL-10 produced by type 2 helper T lymphocytes (Th2) [33]. IL-4 is a type of cytokine that is directly responsible for the production of IgE antibodies and the development of an allergic reaction [34]. The results of studies conducted on animal models have shown that rats delivered by caesarean section exhibit a stronger allergic response to allergens (e.g., ovalbumin—OVA) compared to naturally delivered animals. It has been confirmed that early probiotic intervention with *L. acidophilus* can be highly effective in reducing excessive Th2 immune responses and increasing mRNA expression for tight junction proteins, which enhances allergy prevention in offspring born by caesarean section [33]. Despite evidence from experimental models suggesting an immunomodulatory effect of the mode of delivery, the results of large population studies, such as the Health Nuts study [35] (which analysed a cohort of 2045 infants) indicate that caesarean section—whether planned, emergency or preceded by the onset of labour—is not significantly associated with an increased risk of food allergy [35]. Similarly, a long-term analysis of a cohort of 2114 Japanese children, conducted until the age of 9, did not show a statistically significant association between the mode of delivery and the risk of developing food allergies, bronchial asthma and atopic dermatitis [36]. It should be noted that, compared to humans, the previously discussed laboratory animals (piglets) live in very sterile and controlled conditions that meet so-called SPF (Specified Pathogen Free) standards. Limited exposure to pathogens (e.g., viruses) and microorganisms contributes to a poorer microbiome in laboratory animals and the failure to develop a mature immune system. This may translate into a stronger response to allergens on the one hand, and a weaker Th2-type immune response as a result of probiotic intervention with Lactobacillus acidophilus on the other hand [37]. Moreover, laboratory animals are often deprived of certain factors that burden the body, such as chronic stress, drug use, contact with air pollution and an unbalanced diet based on highly processed products.

An innovative approach to non-pharmacological management of food allergies is the biotechnological enhancement of safety for individuals with allergic hypersensitivity through the production of hypoallergenic food. It has been shown that certain strains of *Lactobacillus* can be used in the fermentation process to reduce the immunogenicity of high-protein raw materials, such as shrimp meat (*Litopenaeus vannamei*). Studies have confirmed that *L. acidophilus* (6005) has the ability to proteolytically degrade both sarcoplasmic and myofibrillar proteins, including the main shrimp allergen—tropomyosin, a 36 kDa muscle protein. SDS-PAGE analysis confirmed that the interaction of *L. acidophilus* leads to enzymatic (proteinase-mediated) degradation of IgE-binding epitopes, which enables the production of hypoallergenic food from shrimps with the total allergenicity of the product reduced by 70% [38].

Studies have shown that a fermented soy drink enriched with two bacterial strains (*L. acidophilus* and *L. plantarum)*, a prebiotic in the form of fructo-oligosaccharides and grape seed extract may be highly effective in inhibiting an excessive, multi-stage immune response to food allergens. It has been confirmed that a multi-component functional product containing the *L. acidophilus* strain can block the degranulation of effector cells (mainly mast cells and basophils), which results in the inhibition of the release of mediators of acute allergic inflammation (such as histamine, tryptase, platelet-activating factor, prostaglandins and leukotrienes) and thus inhibits the occurrence of local and systemic allergic symptoms. The described phenomenon occurs by limiting the influx of Ca^2+^ ions from the outside and the endoplasmic reticulum to effector cells, which directly results in the inhibition of the release of both histamine and other inflammatory mediators (e.g., leukotrienes), but also in the reduction in the level of inflammatory markers such as TNF-α and IL-6 in the gastrointestinal tract. The cited studies also indicate the occurrence of significant synergy between probiotics and phenolic substances contained in the soy and grape seed matrix, which enhances the protective potential of *L. acidophilus* in food allergies [39].

Studies on the use of combined strains of *L. acidophilus* NCFM and *Bifidobacterium lactis* BL-04 together with fructo-oligosaccharides (FOS) have shown that intervention with this type of synbiotic can be highly effective in the treatment of allergic rhinitis and ocular conjunctivitis in patients allergic to birch pollen. It has been shown that four-month supplementation with the described synbiotic leads to a reduction in the total symptom score (TSS) by 80% and the total nasal symptom score (TNSS) by 50%, which confirms that this type of therapy can be effective in both patients with and without birch pollen-induced asthma. The protective effect of the NCFM *L. acidophilus* strain against birch pollinosis has been shown to be primarily due to inhibition of the late-phase reaction, which is an inflammatory response that occurs several hours after contact with the allergen. The NCFM *L. acidophilus* strain may significantly limit cell damage, such as tissue infiltration by eosinophils and T lymphocytes, which accompanies the late-phase reaction (LPR), which follows the immediate early reaction (IER) [40].

Multi-year clinical studies on the use of *L. acidophilus* extract have demonstrated that this probiotic can be highly effective in the treatment of cedar pollinosis, while also serving as an important complement to sublingual immunotherapy (SLIT). Early intervention with *L. acidophilus* extract, initiated during the pre-season, has been shown to stabilize cedar pollen-specific IgE levels and significantly reduce clinical symptoms of allergic rhinitis, such as nasal mucosa swelling. Furthermore, compared to standard immunotherapy, which often requires up to three years of sublingual desensitization (SLIT) to achieve full clinical effectiveness, supplementation with *L. acidophilus* has been associated with a reduction in specific IgE antibody levels already within the first year of treatment [41].

New research indicates that the L-92 strain of *L. acidophilus* may be highly effective in inhibiting both passive (PCA) and active (ACA) cutaneous anaphylaxis, the initial symptom of a severe, systemic allergic reaction manifesting as sudden, intense hives, itching, erythema, and swelling (e.g., of the lips or eyelids) that can lead to life-threatening anaphylactic shock. Oral supplementation of this strain (as a paraprobiotic) has been shown to suppress specific IgE antibody titers, directly inhibiting the mechanisms of cutaneous anaphylaxis. At the tissue level, *L. acidophilus* (strain L-92) reduces the infiltration of mast cells and eosinophils in the mucosa and connective tissue, resulting in the suppression of inflammation induced by environmental allergens such as house dust mites, pollen, and mold spores. *L. acidophilus* has been observed to have the ability to restore cytokine homeostasis by restoring the balance between Th1 and Th2 lymphocytes, resulting in a reduction in the activity of Th2 lymphocytes, which are responsible for the development of various forms of atopic dermatitis [42].

Despite favorable results regarding the effect of *L. acidophilus* on the development of immune tolerance and, consequently, on reducing autoimmune diseases and allergies, previous clinical evidence suggests significant heterogeneity in the body’s response to probiotic supplementation. It was confirmed that early supplementation with *L. acidophilus* (LAVRI-A1) in high-risk neonates (children of mothers with food allergy) at both 6 and 12 months of age did not reduce the incidence of atopic dermatitis (AD), the incidence of which was almost the same as in the placebo group. In the described case, *L. acidophilus* supplementation not only failed to reduce the incidence of atopic dermatitis associated with cow’s milk protein allergy (primarily to casein and β-lactoglobulin), but was also associated with a significant increase in IgE antibody production and an increased risk of allergy to cow’s milk proteins [43]. These results emphasize the need for great caution in routinely recommending probiotic supplementation for the prevention of allergic and autoimmune diseases and suggest that the effect of microbiota modulation on the immune system is strongly dependent on the probiotic strain, the dose used, and individual genetic predispositions determining the body’s response to the presence of a probiotic. The presented results of studies using various strains of *L. acidophilus* also confirm that the probiotic properties of these bacteria are highly specific to a given strain, not the entire species. It should be emphasized that further studies are necessary, which will be conducted on a clinical model involving large numbers of patients, which will confirm or challenge the observations obtained so far on the therapeutic effect of *L. acidophilus* on allergic diseases.

In summary, it should be noted that the effect of *L. acidophilus* in the conditions discussed is primarily based on restoring immune balance by suppressing excessive Th2 lymphocyte responses, reducing the production of IgE and histamine antibodies, and stimulating Treg regulatory lymphocytes and the anti-inflammatory cytokine TGF-β1 they secrete. Additionally, at the cellular level, *L. acidophilus* strengthens the integrity of the intestinal barrier by increasing the expression of tight junction proteins (ZO-1, claudin-1) and strongly inhibits inflammatory processes (reducing the levels of TNF-α and IL-6, among others) by regulating the TLR4/NF-kappa B signaling pathway and by limiting the influx of Ca^2+^ ions into effector cells. Metabolically, the discussed *L. acidophilus* strains contribute to the favorable remodeling of the gut microbiota and the promotion of taxa producing short-chain fatty acids (SCFAs), which are the main energy source for intestinal epithelial cells. Furthermore, based on fermentation processes, *L. acidophilus* can perform proteolytic enzymatic degradation of allergenic proteins to directly remove immunoglobulin E (IgE)-binding epitopes.

Based on the reports discussed, several key strategies for the prevention and treatment of allergic diseases using *L. acidophilus* bacteria can be identified. One of these is supplementing the diet with the probiotic *L. acidophilus* and multi-ingredient functional foods (e.g., fermented soy beverages with grape seed extract), which helps restore immunological balance in food allergies by silencing the Th2 response and sealing the intestinal barrier. Another strategy is to produce hypoallergenic food by using specific strains of *L. acidophilus*, which in the fermentation process lead to proteolytic degradation of allergenic proteins (e.g., tropomyosin in shrimp) and destruction of their IgE-binding epitopes, which significantly reduces the overall allergenicity of the food product. Moreover, an important strategy may also be to support conventional immunotherapies, e.g., sublingual immunotherapy (SLIT), where pre-seasonal implementation of L. acidophilus extract allows for stabilization and significantly accelerates the reduction in specific IgE antibody titers, while effectively complementing standard desensitization.

## 5. *Lactobacillus acidophilus* in Cancer Prevention and Therapy

Research findings indicate that various strains of *L. acidophilus* may play a significant role in cancer prevention and may also support oncological treatment, thereby improving the quality of life of oncological patients. An appropriately tailored medical diet supplemented with *L. acidophilus* strains may reduce the risk of cancer development and support the treatment process, for example by inhibiting the uncontrolled proliferation of cancer cells [44].

### 5.1. The Effect of Lactobacillus acidophilus in Inhibiting the Development of Colon Cancer

Clinical studies conducted on a large group of patients (*n* = 600) have confirmed that there is a close correlation between the composition of the gut microbiota and the pathogenesis of colorectal cancer (CRC). Quantitative real-time PCR (qPCR) analysis showed that healthy individuals have a 3.4-fold higher abundance of *L. acidophilus* in the ileum and large intestine compared to patients with colorectal cancer. At the same time, in people with cancer, the number of *Enterococcus faecalis* is more than twice as high, which indicates a deep dysbiosis accompanying the processes of carcinogenesis in the large intestine [45]. For this reason, the change in the number of *L. acidophilus* can be considered one of the key markers of intestinal homeostasis, which are used both in the prevention and therapy of colon cancer.

**Figure 4:**
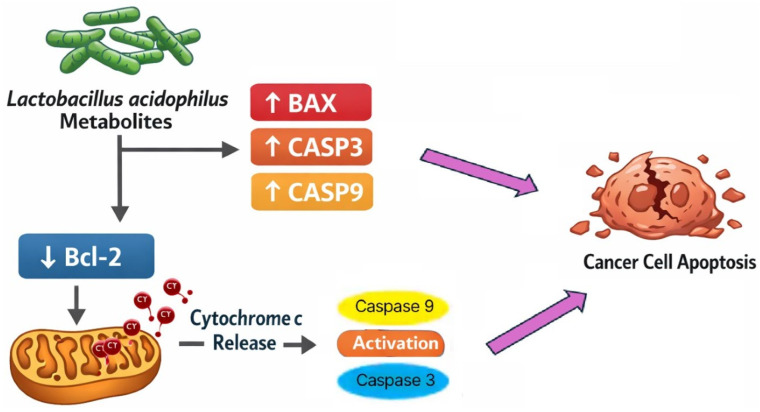
Mechanism of *L. acidophilus* in the epigenetic regulation of colon cancer cells [46].

It has been suggested that *L. acidophilus* may exert anticancer activity through multilevel genetic and epigenetic regulation of colon cancer cells. Studies using HT29 (a human colon cancer cell line) and SW480 (cells isolated from the colon of a patient with Dukes’ type C colon cancer) cell lines have demostrated that metabolites of *L. acidophilus* can induce apoptosis in colon cancer cells by increasing the expression of proapoptotic genes, such as BAX (Bcl-2-associated X protein), CASP3 (Caspase 3 Protein coding gene), and CASP9 (Caspase 9 Protein coding gene), thereby promoting programmed cell death (apoptosis) [46]. At the same time, *L. acidophilus* has been shown to reduce the levels of the antiapoptotic protein Bcl-2 (B-cell lymphoma 2), a key regulator that prevents apoptosis by inhibiting the release of cytochrome c from mitochondria and blocking activation of the caspase cascade (Figure 4) [47]. Moreover, *L. acidophilus* can significantly decrease the expression of matrix metalloproteinases MMP-2 and MMP-9, suggesting strong potential to inhibit metastases and reduce tumor invasiveness (Figure 4) [46]. In addition, *L. acidophilus* may help restore the balance of small non-coding RNAs (miRNAs), upregulating tumor suppressor miR-34 and let7, while downregulating oncogenic microRNAs, such as miR-21 and miR-155, which are closely associated with tumor growth and metastasis [46,48].

It has been confirmed that the secretome, i.e., molecules secreted by the *L. acidophilus* PTCC 1643, can directly inhibit the invasiveness and migration of colon cancer cells. The culture supernatant *L. acidophilus* has also been shown to exert an antiproliferative effect by inhibiting the genetic activity of certain matrix metalloproteinases, such as MMP-9, which, by limiting the degradation of tissue barriers (especially type IV collagen in basement membranes), prevents the remodeling of the extracellular matrix, which is essential for cancer invasion. At the same time, the *L. acidophilus* supernatant induces overexpression of the MMP-12 gene, which is responsible for both the degradation of extracellular matrix proteins and the activation of antiangiogenic pathways, which may lead to the inhibition of tumor growth and the reduction in tumor metastasis [49].

Modern approaches to the prevention and treatment of colorectal cancer (CRC) increasingly incorporate the use of bioactive metabolites of lactic acid bacteria. It has been demonstrated that the exopolysaccharide LA-EPS-20079 (bacterial Exo-Penta-Saccharide) derived from the *L. acidophilus* strain may exhibit potent cytotoxic properties by reducing the synthesis of inhibitors of apoptosis (IAP) proteins. Studies conducted on the CaCo-2 cell line indicate that exopolysaccharides from *L. acidophilus* can reduce the expression of the antiapoptotic gene BCL2 by nearly 3.4-fold and decrease the expression of the Survivin gene, which encodes negative regulatory proteins that prevent apoptotic cell death, by approximately 21.4-fold [50].

An important mechanism underlying the anticancer effects of exopolysaccharides produced by *L. acidophilus* is their strong antioxidant activity. *In vivo* animal studies on rats have shown that administration of exopolysaccharides (derived from *L. acidophilus*) at doses of 200–400 mg/kg body weight can lead to a nearly 50% reduction in the number of 1,2-dimethylhydrazine (DMH)-induced neoplastic polyps formed in the large intestine. A key element of the bioactivity of *L. acidophilus* exopolysaccharides is their ability to restore redox homeostasis by significantly enhancing the activity of antioxidant enzymes such as SOD, CAT, and GPx, as well as regenerating vitamin C and glutathione (GSH) reserves, which are significantly depleted during neoplastic processes in colonic tissue. Consequently, *L. acidophilus* exopolysaccharides inhibit lipid peroxidation processes, which are largely responsible for histological changes in the colonic mucosa and intestinal neoplasia, i.e., abnormal cell proliferation in the intestines (in the form of adenomas or polyps) [51].

Results from *in vivo* studies in a rat model indicate that taking *L. acidophilus* may be highly effective in the treatment of colon cancer, both as a monotherapy and in combination with pterostilbene [52]. Pterostilbene is a natural polyphenolic antioxidant, structurally similar to resveratrol, but with higher bioavailability and a longer duration of biological activity in the body [53]. It has been shown that the administration of a probiotic bacterium, *L. acidophilus*, together with pterostilbene can significantly inhibit the development of early cancerous changes resulting, for example, from the action of carcinogenic substances such as 1,2-dimethylhydrazine (1,2-DMH). The synergistic effect of *L. acidophilus* and pterostilbene leading to the limitation of colon carcinogenesis may result from beneficial changes in the intestinal microbiota and from the strong antioxidant properties of polyphenolic substances from the stilbene group [52].

It has been shown that taking *L. acidophilus* combined with calcium citrate and Moringa oleifera (*Moringa Oleifera* Lam.) extract can lead to a significant reduction in the number of aberrant crypt foci (ACF), which are an early marker of colon neoplasia. Combined supplementation with *L. acidophilus*, calcium citrate, and hydroalcoholic extract from Moringa oleifera leaves has been shown to not only inhibit local intestinal carcinogenesis but also prevent hepatotoxicity induced by carcinogens (such as 1,1-dimethylhydrazine hydrochloride), as confirmed by maintaining physiological levels of markers of liver and biliary damage, such as aspartate aminotransferase (AST), alanine aminotransferase (ALT), and markers of kidney damage, such as urea and creatinine. Additionally, combined supplementation with the above-mentioned ingredients leads to the regeneration of liver histoarchitecture and inhibition of mononuclear cell infiltration, dysplastic changes in hepatocytes, such as hyperchromasia (increased color of the cell nucleus) or enlargement of cell nuclei. Combined supplementation with *L. acidophilus*, calcium citrate, and hydroalcoholic extract from Moringa oleifera leaves has been shown to not only inhibit local intestinal carcinogenesis, but also prevent carcinogen-induced hepatotoxicity, as evidenced by maintained physiological levels of liver injury markers (aspartate aminotransferase (AST), alanine aminotransferase (ALT), and kidney damage markers (urea and creatinine). Furthermore, combined supplementation with these ingredients leads to the regeneration of liver histoarchitecture and inhibition of dysplastic changes in hepatocytes, such as hyperchromasia (increased nuclear staining) and enlarged nuclei [54].

It has been confirmed that the role of *L. acidophilus* in the chemoprevention of colon cancer also includes advanced regulation of metabolic homeostasis and receptor gene expression. Studies in an animal model (BALB/c mice), where colon cancer was induced with azoxymethane, demonstrated that long-term supplementation with *L. acidophilus* can lead to significant improvements in lipid profiles, manifested by reduced triglyceride and LDL (low-density lipoprotein) cholesterol levels in the blood. This indicates that this probiotic may significantly limit the availability of lipids that could be used by tumors for cell membrane construction and cell proliferation. Simultaneously, *L. acidophilus* use has been associated with a reduction in alkaline phosphatase (ALP) activity, suggesting systemic hepatoprotective effects and reduced hepatic carcinogen toxicity [55]. Probiotic intervention has been confirmed to induce significant suppression of the leptin receptor (LPR) and vitamin D receptor (VDR) gene expression at the molecular level. Leptin can lead to increased expression (overexpression) of sirtuin 1 (SIRT1), which counteracts cell aging and apoptosis. This leads to leptin stimulation promoting cancer cell migration and invasion (HCT-116) and tumor growth, particularly in aging- and obesity-related tumors [55,56]. Given that leptin overexpression promotes cancer cell proliferation and survival via adipokine-stimulated signaling pathways, *L. acidophilus* may be a significant oncostatic factor in colorectal cancer [55]. At the same time, the increased expression of the vitamin D receptor gene observed with *L. acidophilus* use may reflect a probiotic-induced enhancement of mucosal immunity or inhibition of fibroblasts in the colon cancer stroma via the active form of vitamin D, calcitriol [57]. It has been confirmed that calcitriol inhibits the activity of the Wnt/β-catenin signaling pathway, thereby limiting proliferation in the colon mucosa [56].

*In vivo* studies using an animal model (carcinogenic processes induced by subcutaneous administration of 1,2-dimethylhydrazine hydrochloride to rats) have shown that long-term oral supplementation with the *L. acidophilus* CGMCC 878 strain leads to attenuated colon tumor development, accompanied by a significant reduction in the number of pathogenic bacteria such as *Ruminococcus obeum*, *Clostridium thermocellum*, *Bacteroides vulgates*, *Mycoplasma leachii*, and *Porphyromonas asaccharolytica*, and an increase in the number of beneficial bacteria such as *Lactobacillus reuteri*. It has been proven that long-term administration of *L. acidophilus* can significantly reduce the expression of β-glucuronidase, an enzyme that metabolizes and reactivates carcinogenic substances into even more toxic forms, which can cause extensive intestinal damage [58].

In the search for innovative strategies to prevent colon cancer, increasing attention has been paid to plant-based dairy alternatives, such as pistachio milk, which, when fermented with selected combinations of probiotic bacteria, may contribute to reducing cancer risk. Studies have shown that, in pistachio milk (a non-dairy drink made from pistachios) enriched with 4% inulin, the *L. acidophilus* strain can degrade inulin-type fructans into short-chain inulin fractions, which can then be metabolized into acetate by *Bifidobacterium* strains, such as *B. bifidum*. The acetate-rich fermented pistachio milk produced under these conditions exhibits cytotoxic effects and induce apoptotic cell death in human colon carcinoma cell lines (Caco-2) by disrupting microtubules and damaging the cell nucleus via the key cysteine protease caspase-3 [59]. For this reason, products such as fermented pistachio milk are becoming a new therapeutic food used in the prevention and treatment of colon cancer.

It has been proven that the *L. acidophilus* strain, in combination with *Bifidobacterium bifidum*, may be particularly useful in the treatment of colon cancer, along with substances such as nisin (a polycyclic peptidnisin with anticancer properties), 5-fluorouracil (used alone or in combination therapy in the treatment of malignant tumors), and selenium (which inhibits the modification of proteins responsible for the carcinogenesis process). Encapsulating these substances in thiolated chitosan nanoparticles conjugated with folic acid (in the form of the N/5FU/Se@FTCsNPs formulation) allows their release only in the alkaline environment of the colon, without releasing these substances in the acidic environment of the stomach. Nisin, 5-fluorouracil, and selenium, in combination with probiotic strains, have been confirmed to selectively inhibit colon cancer cell proliferation *in vitro* (CT26 cells) (IC_50_: 1.57 µg/mL), with minimal impact on healthy intestinal cells. It has been shown that one of the general mechanisms of apoptosis of colon cancer cells induced by nanoparticles (containing nisin, 5-fluorouracil, selenium, and probiotic strains) is the overproduction of free radicals and the resulting strong oxidative stress. It has been confirmed that combining these active substances with probiotics results in increased expression of tumor suppressor genes, such as PTEN (phosphatase and tensin homolog, a negative regulator of the PI3K/AKT/mTOR pathway that inhibits cancer cell growth and division) and the CASP9 gene (caspase 9), which encodes a key protein initiating the intrinsic pathway of cancer cell apoptosis. Simultaneously, the presence of *L. acidophilus* and *B. bifidum* enhances the suppression of pro-angiogenic and proliferative pathways, such as mTOR and VEGF-α. Additionally, the probiotic bacteria used in the studies may strengthen the intestinal barrier by increasing the expression of the Mucin 2 gene, which helps reduce the overall toxicity of chemotherapy used in colorectal cancer, manifested by diarrhea and weight loss [60].

### 5.2. The Effect of Lactobacillus acidophilus in the Treatment of Liver Cancer

**Figure 5:**
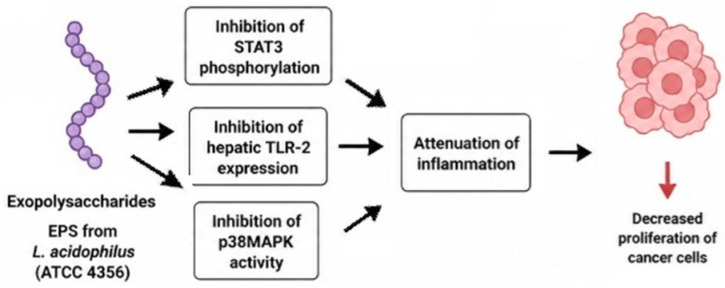
Exopolysaccharides produced by *L. acidophilus* limit the expression of TLR-2 and inhibit the phosphorylation of STAT3 and p38MAPK kinase, leading to attenuation of inflammation, which is responsible for hepatocarcinogenesis [61].

Exopolysaccharides synthesized by *L. acidophilus* ATCC 4356 have been shown to exert a multifaceted oncostatic effect on hepatocellular carcinoma (HCC), the most common form of primary liver cancer originating from hepatocytes. These exopolysaccharides can effectively inhibit hepatocarcinogenesis induced by the synergistic action of chemical (diethylnitrosamine) and radiation (gamma radiation) factors, as evidenced by reduced activity of liver and bile duct damage markers, such as alanine aminotransferase (ALT) and gamma-glutamyltranspeptidase (GGTP), as well as mitigation of oxidative stress, indicated by decreased levels of free malondialdehyde (MDA) in the blood. In a rat model study, it was confirmed that exopolysaccharides derived from *L. acidophilus* ATCC 4356 limit the overexpression of the liver receptor TLR-2 and inhibit the phosphorylation of STAT3 protein and p38MAPK kinase, thus leading to a reduction in the pro-inflammatory state that determines the proliferation of cancer cells. Exopolysaccharides produced by *L. acidophilus* ATCC 4356 reduce the levels of pro-inflammatory cytokines, such as interleukin 17 (IL-17) produced by Th17 lymphocytes, and the level of the collagen-stimulating protein TGF-beta 1, which suggests that the discussed probiotic may play (especially in advanced stages) an important role in inhibiting the process of liver fibrosis and the progression of malignant neoplastic lesions (Figure 5) [61].

It has been confirmed that *L. acidophilus* may exert anticancer effects by modulating the gut–liver axis. Results from studies in mouce models indicate that a decrease in intestinal *L. acidophilus* is an important marker of hepatocellular carcinoma (HCC) progression, while a re-increase in the number of this strain may cause a strong oncostatic effect. Reports indicate that the oncostatic mechanism is closely related to the production of valeric acid by *L. acidophilus*, a short-chain fatty acid that acts as a ligand for the GPR41 and GPR43 receptors on the surface of hepatocytes. As a result of activation of GPR41 and GPR43, the signaling pathway involving Rho-GTPase proteins (Ras homologues) is inhibited, the excessive overactivity of which is responsible for the growth, survival and migration of cancer cells. Inhibition of Rho-GTPase activity leads to the activation of the p38 mitogen-activated protein kinase (MAPK) signaling pathway, resulting in reduced cancer cell proliferation and increased apoptosis. It has been shown that, in addition to inhibiting carcinogenesis, supplementation with *L. acidophilus* also strengthens the intestinal barrier and reduces metabolic liver inflammation, which makes this strain an important element in both supportive therapy and the prevention of liver cancer [62].

Evidence suggests that *L. acidophilus* (CICC 20244 strain) may be an important factor in enhancing anticancer oncolytic virotherapy for the treatment of hepatocellular carcinoma. In an animal model study (female C57BL/6J mice), it was shown that viral therapies using the VSVΔ51 virus (a mutated oncolytic form of vesicular stomatitis virus (VSV)) can simultaneously induce intestinal dysbiosis. VSVΔ51 has been shown to inhibit the expression of the SLC20A1 protein (sodium-phosphate cotransporter, also known as PiT-1) in the intestine, which acts as a docking element for the three-domain *L. acidophilus* cell wall protein CdpA. This causes *L. acidophilus* to lose its ability to adhere to the intestinal epithelium and colonize the gut. In turn, restoring the homeostasis through supplementation with *L. acidophilus* enhances intestinal barrier integrity, increases the number of cytotoxic CD8+ T lymphocytes (producing perforin, granzyme B, IFN-γ and TNF-α), and reduces the number of dysfunctional CD8^+^ T lymphocytes in the tumor microenvironment of liver cancer, thereby enabling a more effective immune response against the disease [63,64].

Animal model studies on rats have indicated that *L. acidophilus* can effectively support the treatment the ulcerative colitis, which significantly increases the risk of developing colon cancer. By influencing mRNA expression, *L. acidophilus* has been shown to reduce the levels of key pro-inflammatory cytokines involved in the pathogenesis of ulcerative colitis, including TNF-alpha, IL-1beta, IL-6, and IFN-gamma. Simultaneously, this probiotic significantly increases the levels of anti-inflammatory cytokines, such as interleukin 10 (IL-10). It has been found that *L. acidophilus* can also influence the levels of microRNAs (miRNAs), short, single-stranded RNA molecules that do not encode proteins but act as potent regulators of gene activity. *L. acidophilus* can restore normal levels of miR-1, miR-let-7d, and miR-99a, which inhibit cell division and cancer cell migration, thereby limiting metastasis. At the same time, *L. acidophilus* can reduce the levels of miR-155 (an oncomiR involved in macrophage accumulation and chronic inflammation), which ultimately leads to attenuation of excessive inflammation in the intestines and reduced cancer progression [65].

### 5.3. The Effect of Lactobacillus acidophilus in the Treatment of Cervical Cancer

It has been shown that *L. acidophilus* metabolites may be adjuvants that effectively support chemotherapy for cervical cancer. It has been shown that the culture supernatant of *L. acidophilus* can induce apoptosis in CaSki cells by increasing the activity of the proapoptotic gene BAX and reducing the expression of the antiapoptotic gene BCL2. Consequently, the expression of caspase-3 increases, which leads to protein proteolysis and morphological changes in the cancer cell and ultimately to its death. Additionally, the culture fluid from *L. acidophilus* selectively reduces the expression of the matrix metalloproteinase MMP9 gene, which limits the ability of cancer cells to invade tissues and form metastases [66].

Studies have shown that intracellular protein fractions derived from the Indonesian strain of *L. acidophilus* (IIA-2B4) (isolated from raw beef) have also strong inhibitory properties against a cervical cancer cell line (HeLa cells). The results of confocal microscopy showed that the culture extract of *L. acidophilus* caused deformation and disintegration of cancer cells (HeLa), with an effect nearly three times stronger than that of *L. plantarum* [67].

Probiotic therapy with *L. acidophilus* has been shown to be useful adjuvant treatment option for cervical cancer patients, improving tolerance to the side effects of pelvic radiotherapy, such as radiation-induced diarrhea (RID), which affects up to 80% of treated women. Randomized clinical trials confirm that supplementation with the *L. acidophilus* LA-5 strain, in combination with *Bifidobacterium animalis* BB-12, is effective in reducing RID incidents from 82.1 to 53.8%. *L. acidophilus* has been shown to not only significantly reduce the severity of radiation-induced diarrhea, but also significantly reduce abdominal pain associated with cervical cancer. *L. acidophilus* may have supportive effects on cancer radiotherapy, including: by stimulating the body’s production of lactase, which supports the digestion of lactose, and whose activity is reduced or completely inhibited due to damage to the intestinal villi as a result of the use of high-energy ionizing radiation (X or gamma radiation) used to destroy cancer cells [68].

It has been shown that cell-free culture supernatants of *L. acidophilus* (LACFS strain) can exert potent cytotoxic and oncostatic effects on cervical cancer cells. Studies using a human cervical cancer cell line (SiHa cell line) confirmed that *L. acidophilus* is particularly effective against cancer caused by the human papilloma virus (HPV), chronic infection with which (primarily HPV types 16 and 18) is the leading cause of cervical cancer in women. It has been confirmed that *L. acidophilus* metabolites induce cell death through induced shrinkage, membrane blebbing, and loss of adhesion to the substrate, which is crucial for cancer cell growth and tumor formation. Furthermore, *L. acidophilus* metabolites can significantly inhibit the ability of cancer cells to migrate. In clinical practice, this means that the action of *L. acidophilus* may significantly reduce the risk of cancer metastasis to other organs [69].

### 5.4. The Effect of Lactobacillus acidophilus in Breast Cancer Immunotherapy

It has been confirmed that the use of *L. acidophilus* as a component of acidophilic milk, in combination with propolis extract, may represent a promising approach in the development of supportive therapies for breast cancer. Studies conducted on an animal model (Balb/c mice injected subcutaneously with 4T1 mouse mammary cancer cells) have shown that this combination exerts a strong synergistic effect, resulting in a reduction in tumor volume of up to 63.39%. The observed effect was significantly higher than the effect of both factors used as monotherapy, where acidophilic milk alone (containing the *L. acidophilus* LA-5 strain) reduced the tumor volume by 28.29%, while propolis extract alone reduced the tumor volume by 59.16%. It has been suggested that the anticancer mechanism of the combination of *L. acidophilus* and propolis extract is based, in part, on the induction of apoptosis in cancer cells, mediated by the stimulation of splenocyte proliferation in the spleen. These splenocytes—primarily T and B lymphocytes, as well as macrophages and dendritic cells—play a key role in immune recognition and the production of specific antibodies. On the other hand, combined therapy with *L. acidophilus* and propolis extract significantly induces the secretion of interferon gamma (IFN-γ), a key cytokine (produced by T lymphocytes and NK cells) responsible for the synthesis of other cytokines, such as dichotomous interleukin 2, pro-inflammatory interleukin 6, and tumor necrosis factor TNF-α. This ultimately leads to the mobilization of a cellular response and apoptosis of cancer cells within the breast tumor [70].

Modern approaches to breast cancer treatment increasingly include the use of *L. acidophilus* as a component of adjunctive therapy aimed at increasing the therapeutic index of cytostatic drugs used in breast cancer treatment. Studies using a breast cancer cell model (4T1 cells) have shown that co-administration of *L. acidophilus* (in combination with *L. casei* and vitamin D3) during doxorubicin treatment can lead to a synergistic reduction in tumor mass and volume. The mechanism of this interaction has been suggested to be based on increased activity of the proapoptotic genes Bax and caspase 3, while simultaneously suppressing the antiapoptotic gene for the Bcl-2 protein. Additionally, *L. acidophilus* protects intestinal tissues, significantly reducing the cytotoxic side effects of chemotherapy used in breast cancer [71].

The oncoprotective (anticarcinogenic) properties of *L. acidophilus* also include its ability to directly neutralize highly carcinogenic mycotoxins, such as aflatoxin M1 (a group 2B carcinogen), which is a common contaminant in human milk. Studies have shown that live *L. acidophilus* cells at a concentration of 10^8^ CFU/mL (added to human milk from breastfeeding mothers) are able to permanently bind up to 87.51% of aflatoxin M1 molecules, effectively removing this carcinogen from human milk [72].

It has been confirmed that the effects of traditionally used oncological drugs, such as SERMs (selective estrogen receptor modulators), like tamoxifen, may involve not only their effect on estrogen receptors, but also their influence on the composition of microorganisms inhabiting breast tissue. It has been shown that hormonal therapy with tamoxifen can induce a profound remodeling of the local microbiome of breast tissue, favoring the growth of *Firmicutes* bacteria, including bacteria expressing lipoteichoic acid (LTA), a component of the cell walls of Gram-positive bacteria, such as *L. acidophilus*. This phenomenon may directly inhibit the process of carcinogenesis, as evidenced by the observed negative correlation between the presence of LTA-positive bacteria (including *L. acidophilus*) and the expression of the nuclear protein Ki67, a key marker of cell proliferation in human breast cancer. In animal model studies (B6.MMTV-PyMT mice), direct intra-nipple injection of probiotics directly into the duct openings of the nipple reduced tumor formation by altering the expression of metabolic genes and limiting cancer cell proliferation. Additionally, substances secreted by *L. acidophilus* may selectively damage estrogen receptor-positive (ER+) breast cancer cells (by disrupting their energy metabolism), while not harming healthy epithelial cells of the ducts and lobules of the mammary gland. This suggests that the mammary gland microbiome may act as an active intermediary in the action of antiestrogen drugs, and targeted modulation of this microbiome may be a strategy to reduce the risk of breast cancer progression [73].

**Figure 6:**
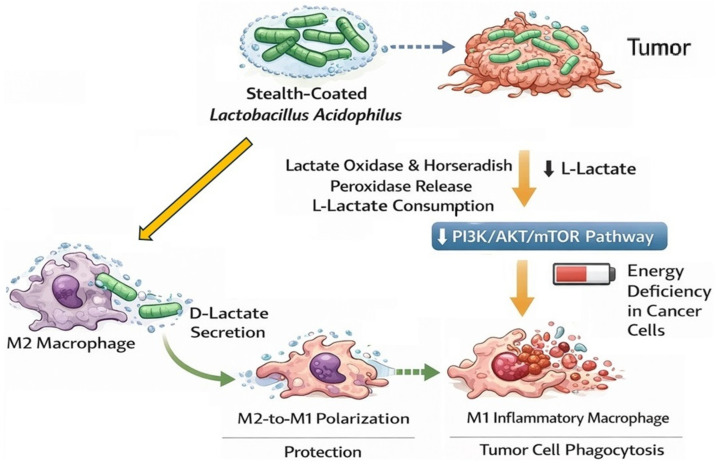
Mechanism of cancer cell homeostasis disruption by stealth polymer-coated *Lactobacillus acidophilus* (LH@LA) [74].

Modern therapeutic strategies increasingly employ advanced genetic and chemical engineering to transform *L. acidophilus* into an organ for delivering therapeutic substances directly to the tumor microenvironment. One example is the LH@LA system, in which *L. acidophilus* cells are coated with a protective “stealth” polymer layer, that enables them to effectively avoid phagocytosis by macrophages. This modification facilitates deeper tumor penetration and allows for the sustained release of encapsulated enzymes, such as lactate oxidase and horseradish peroxidase. These enzymes catalyze the depletion of L-lactate in tumor tissues, a key metabolite associated with immunosuppression. Reduction in L-lactate levels alleviates immunosuppressive conditions and enhances the efficacy of anticancer therapies. Furthermore, the degradation of L-lactate has been shown to inhibit the PI3K/AKT/mTOR signaling pathway, contributing to metabolic stress and energy deficiency in cancer cells. Concurrently, *L. acidophilus* may promote the polarization of macrophages from the anti-inflammatory M2 phenotype to the pro-inflammatory M1 phenotype, partly through the production of D-lactate (Figure 6). M1 macrophages exhibit enhanced anti-tumor activity via phagocytosis and cytotoxic mechanisms. Overall, these findings highlight the potential of engineered *L. acidophilus* as a novel therapeutic platform for modulating the tumor microenvironment and improving anticancer treatment outcomes [74].

Recent approaches to using *L. acidophilus* strains in oncology extend beyond their effects on the immune system and immune cell activity. It has been shown that *L. acidophilus* may constitute a highly efficient production factor in the process of green synthesis of nanomaterials with anticancer properties. Recent reports indicate that *L. acidophilus* enables the production of stable silver nanoparticles (AgNPs) with a crystalline structure that is naturally stabilized by bacterial proteins and secondary metabolites. Silver nanoparticles produced by *L. acidophilus* (in the presence of AgNO_3_) have been confirmed to exhibit significant cytotoxic potential against key malignant tumor cell lines, including colon (Caco), lung (A549), and liver (HepG2) cancers. Spherical nanosilver structures of 19–25 nm in size produced by *L. acidophilus* have proved to exhibit strong selective cytotoxicity against many cancer cell lines, particularly against colorectal adenocarcinoma (Caco-2). Additionally, probiotic-produced bionanoparticles exhibit high antimicrobial activity, particularly against *Escherichia coli* and *Salmonella enterica*, indicating a multifaceted therapeutic effect of the products of microbial biotransformation carried out by *L. acidophilus* [75]. Table 2 presents the effects of different *L. acidophilus* strains in reducing allergic and neoplastic diseases.

Although the described *in vitro* and *in vivo* animal studies suggest promising anticancer activity of *L. acidophilus* and its metabolites, rigorous and large-scale clinical trials in humans are still lacking. An important question that remains to be answered is the attenuating effect of probiotics on drugs used in modern cancer therapy. Based on studies on a preclinical model (mice), it has been shown that taking probiotics (in the form of *Lactobacillus* and *Bifidobacterium*) with the diet may impair the body’s response to therapy based on monoclonal antibodies (anti-PD-1) directed against programmed death molecules (PD-1) located on the surface of cytotoxic T lymphocytes (which lymphocytes are capable of releasing cytotoxic proteins, such as perforins and granzymes, inducing apoptosis of cancer cells). By weakening the activity of anti-PD-1 monoclonal antibodies, *L. acidophilus* prevents the blocking of the immune checkpoint in the form of the PD-1 molecule, which causes anti-tumor, interferon-γ-positive T lymphocytes (CD8+) not to secrete the cytokines IFN-γ and TNF-α and thus loses the ability to destroy cancer cells in the tumor microenvironment. For this reason, taking probiotic supplements during cancer therapy (e.g., those available to patients over the counter) should be preceded by a detailed oncological medical consultation to exclude the possibility of reducing the effectiveness of cancer therapies [80,81].

In summary, it should be noted that at the molecular level, *L. acidophilus* bacteria exhibit potent anticancer activity primarily by inducing apoptosis, which is achieved by increasing the expression of proapoptotic genes (such as BAX, CASP3, and CASP9) and simultaneously reducing the activity of antiapoptotic proteins and genes, including Bcl-2 and survivin. Additionally, various *L. acidophilus* strains limit the invasiveness and metastatic potential of cancer cells by inhibiting the expression of specific matrix metalloproteinases (e.g., MMP-2 and MMP-9) and by favorably regulating microRNA levels, which involves the stimulation of tumor suppressors (e.g., miR-34) while simultaneously silencing oncogenic microRNA variants (e.g., miR-155, miR-21). Molecular effects also include suppression of pro-inflammatory and proliferative pathways (such as PI3K/AKT/mTOR, Rho-GTPase, and STAT3) and reduction in pro-inflammatory cytokines (e.g., TNF-alpha, IL-17, IL-6) in favor of increasing the anti-inflammatory interleukin 10 (IL-10). Metabolically, L. acidophilus protects cells against cancer through potent antioxidant activity and restoring redox homeostasis, which occurs by enhancing the activity of antioxidant enzymes (including SOD, CAT, GPx), regenerating glutathione and vitamin C, and inhibiting harmful lipid peroxidation. Furthermore, *L. acidophilus* bacteria influence systemic metabolism by producing bioactive exopolysaccharides and short-chain fatty acids (e.g., valeric acid, which acts as a ligand for the GPR41 and GPR43 receptors), as well as improving the lipid profile by lowering triglyceride and LDL cholesterol levels. The metabolic effects of *L. acidophilus* also include reducing the activity of enzymes that increase the toxicity of carcinogens, such as β-glucuronidase, which directly protects tissues from damage and neoplastic transformation.

Based on the reports discussed, several key strategies for the prevention and treatment of cancer using *L. acidophilus* can be identified. One such strategy is the use of various *L. acidophilus* strains in dietary supplementation through the implementation of specific functional foods (such as fermented pistachio milk), which allows for the restoration of gut microbiota homeostasis and, consequently, inhibition of cancer cell proliferation. Combination therapies, which combine the simultaneous use of *L. acidophilus* with antioxidants (e.g., pterostilbene), plant extracts (e.g., from Moringa oleifera leaves), or chemotherapeutic agents encapsulated with probiotics in nanoparticles (e.g., chitosan), may be an important strategy. This increases the therapeutic efficacy of cytotoxic drugs while limiting their systemic and organ toxicity. *L. acidophilus* also demonstrates high potential as an adjuvant in conventional oncological treatment, improving patient tolerance to radiotherapy. Modern strategies also focus on direct modulation of the tissue microbiome (e.g., via intraductal injections in the mammary gland) and the use of genetic and chemical engineering (e.g., the LH@LA system), in which polymer-coated *L. acidophilus* bacteria serve as a platform for delivering enzymes that remove immunosuppressive metabolites directly to the tumor environment. Additionally, *L. acidophilus* bacteria can be used in chemoprevention to directly neutralize highly carcinogenic mycotoxins in the body and can be used in bionanotechnology as an efficient agent in the green synthesis of silver nanoparticles, which exhibit strong selective cytotoxic activity.

## 6. Other Therapeutic and Protective Effects of *Lactobacillus acidophilus*

It should be emphasized that the examples discussed herein do not represent all known properties and effects of *L. acidophilus* related to the prevention of cancer and allergic diseases. A promising avenue for utilizing the cytostatic properties of *L. acidophilus* is its use in the treatment of aggressive oral cancers, such as squamous cell carcinoma (SCC), a malignant skin tumor of epithelial origin that often metastasizes to distant organs. Recent evidence indicates that *L. acidophilus* exhibits potent antiproliferative properties, significantly reducing the viability of human oral squamous cell carcinoma cell lines in the head and neck region. It has been confirmed that the mechanism of the anticancer effect of this probiotic is largely based on the induction of apoptosis caused by the activation of the newly discovered cytokine TRAIL (TNF-Related Apoptosis Inducing Ligand), which was also shown to have low toxicity towards healthy, non-transformed epithelial cells [76].

*L. acidophilus*, through their anti-inflammatory and antigenotoxic effects, have also been shown to be effective in the prevention and treatment of Barrett’s esophagus, which is considered a precancerous condition that significantly increases the risk of developing esophageal adenocarcinoma. Cell-free supernatant from *L. acidophilus*, as well as live bacteria, have been shown to reduce DNA damage and chronic inflammation in esophageal tissues by inhibiting the excessive activation of the nuclear transcription factor NF-κB, a potent regulator of the inflammatory response in the body [77].

It has also been confirmed that *L. acidophilus* may have antiproliferative and antiangiogenic effects on gastric cancer cells. It may reduce the viability of cancer cells and reduce the mass of tumors. Probiotic bacteria administered in combination with celecoxib (a nonsteroidal anti-inflammatory drug, a selective cyclooxygenase-2 (COX-2) inhibitor) have been shown to synergistically reduce COX-2 expression, which consequently leads to inhibition of tumor blood vessel formation (angiogenesis) and induction of cancer cell death (apoptosis) in gastric tumors [78].

Research indicates that the AJ2 strain of *L. acidophilus* can exert an immunomodulatory effect on the secretion of interferon gamma (IFN-γ) from immune cells, leading to reduced bone tumor growth and metastasis. Oral supplementation with this probiotic has been shown to prevent tumor-induced bone loss (bone resorption) in osteolytic tumors [14].

The immunomodulatory effects of *L. acidophilus* may also be manifested in regulating the gut–skin axis, providing protection against UVB-induced skin cell damage. Oral supplementation with the paraprobiotic tyndallized *L. acidophilus* has been documented to effectively inhibit skin cell photoaging by suppressing matrix metalloproteinases (MMP-1 and MMP-9) and inhibiting the mitogen-activated kinase (MAPK) signaling pathway, which results in inhibiting cancer cell proliferation and migration. *L. acidophilus* has also been shown to have a positive effect on the skin by limiting transepidermal water loss (TEWL) and by protecting and stimulating collagen fiber synthesis, which helps maintain the integrity of the skin’s water-lipid barrier [79].

It has been confirmed that *L. acidophilus* can also play an important role in the microbiological safety of food by preventing the development of harmful pathogenic microorganisms that pose a risk to consumer health. One of the significant threats in the production of meat, fish and dairy products is the contamination of food raw materials with the *Listeria monocytogenes*, which can lead to the development of listeriosis—a disease that poses a serious risk to pregnant women, newborns and immunocompromised individuals [82]. Studies have shown that many strains of *L. acidophilus* have a strong antagonistic potential against *Listeria monocytogenes*, constituting a potential element of food safety and listeriosis prevention [83]. *In vivo* studies using an animal model (male Rex rabbits) showed that supplementation with *L. acidophilus* strain ACCC11073 (at a dose of 10^8^ CFU/kg of diet) significantly reduced the *Listeria monocytogenes* titre in organs such as the liver, spleen, lymph nodes, and caecum to a level comparable to that observed during antibiotic therapy with enrofloxacin. The mechanism of action of *L. acidophilus* was confirmed to be based on direct inhibition of bacterial growth, but also on modulation of the host immune response by reducing the expression of genes from the MAPK (Mitogen-Activated Protein Kinase) kinase family and reducing the levels of oxidative stress markers and pro-inflammatory cytokines such as IL-1β, IL-6, and TNF-α [83]. It is emphasized that the ability to maintain the growth potential of *L. monocytogenes* at a level no higher than the threshold (≤0.5 log10 cfu/g) is particularly important in the production of products with a high risk of contamination, such as marinated RTE-fish (RTE, ready-to-eat products) or RTE-meat products. The use of *L. acidophilus* as a feed additive or a functional food ingredient may represent an effective strategy to reduce the risk of *L. monocytogenes* infection while improving the overall microbiological quality and shelf life of food products [82,83].

## 7. Contraindications to the Use of Probiotics of the *Lactobacillus* Genus

Clinical observations indicate that although lactic acid bacteria, including *L. acidophilus*, constitute a dominant component of the body’s microbiota and many fermented foods, they can also cause dangerous infections, primarily in individuals with developing metabolic syndrome (e.g., obesity and concomitant high blood pressure, carbohydrate or lipid metabolism disorders) and in immunocompromised individuals. It has been suggested that factors associated with an increased risk of *Lactobacillus* infection include the presence of prosthetic heart valves, previous dental procedures, and poor oral health, including dental caries [84].

The analysis and characterization of the virulence of various strains of the *Lactobacillus* genus causing infections have shown that the factor that promotes the pathogenicity of this type of bacteria in immunocompromised individuals is the increased ability to form a bacterial biofilm (bacterial membrane), which is formed at the interface between cells and tissues and the air [85].

Clinical reports indicate that *Lactobacillus* may pose a particular threat to patients with endocarditis following prosthetic heart valve implantation, causing mortality rates of 8.3% to 10%. A similarly high risk of death from *Lactobacillus* infection has been observed in immunosuppressed cancer patients and organ transplant recipients, particularly when comorbidities such as diabetes are present [86]. Therefore, methodical genome sequencing to precisely assess the virulence of probiotics and analysis of their genetic stability (to exclude genetic mutations affecting virulence) could allow for excluding dangerous probiotic variants from use and offering only safe and health-beneficial *Lactobacillus* spp. variants for use. Traditionally used fermented dairy products, such as yogurt, buttermilk, or kefir, which are a potential source of lactic acid bacteria in the diet, should also be subject to special virulence assessment, which would improve the safety of these products for people with reduced immunity [84]. Therefore, to reduce the risk of infection, regular testing of the genetic stability of *Lactobacillus* bacteria (e.g., whole genome sequencing (WGS)) should be performed to ensure that only non-pathogenic variants of *Lactobacillus* probiotics are administered to people with metabolic syndrome and immunocompromised individuals.

## 8. Conclusions

Based on the review of research literature from 2019–2026, *L. acidophilus* can be considered a multifaceted element in the prevention and treatment of cancer and allergic diseases. Its effects are manifested at three interrelated levels: molecular, metabolic, and nanotechnological.

First, it should be emphasized that *L. acidophilus* acts as a precise stimulator of the immune and signaling response. By modulating, among others, the MAPK pathway and silencing the IL6-JAK-STAT3 axis, numerous *L. acidophilus* strains can reprogram the tumor microenvironment, promoting the anti-tumor polarization of anti-inflammatory M2 macrophages into pro-immunogenic M1 macrophages and increasing the infiltration of cytotoxic CD8+ lymphocytes into tumor tissues, where they directly destroy tumor cells. The ability of *L. acidophilus* to induce immunogenic cell death (ICD) also makes this probiotic an important component of modern anticancer immunotherapies using oncolytic viruses (e.g., VSVΔ51). By stimulating the production of anti-inflammatory interleukins (e.g., IL-10) and inducing regulatory T lymphocytes (Tregs), *L. acidophilus* can effectively inhibit the inflammatory cascade and reduce the levels of specific IgE antibodies, thereby alleviating the symptoms of asthma, atopic dermatitis or food allergies. *L. acidophilus* may also restore immune balance in allergic diseases by suppressing the Th2 response and inducing Treg lymphocytes and the anti-inflammatory cytokine TGF-β1, while simultaneously strengthening the intestinal barrier (by upregulating ZO-1 and claudin-1 proteins).

Secondly, it should be noted that the metabolism of *L. acidophilus* is a unique source of bioactive oncostatic postbiotics. The production of short-chain fatty acids (especially valeric acid) demonstrates a broad protective effect on organs such as the liver, where, through GPR41/43 receptors, dysfunction of the Rho-GTPase pathway, which can become oncogenic and lead to neoplastic transformation, is inhibited. At the same time, *L. acidophilus* plays a dual protective role, consisting, on the one hand, of eliminating infectious pathogens that increase the susceptibility of host cells to mutations, and, on the other hand, of inducing direct cytotoxicity against cancer cells (e.g., colon). In turn, in the area of allergy prevention, *L. acidophilus* strains favorably change the profile of the intestinal microbiota, leading to an increase in its diversity and stimulating the multiplication of taxa producing short-chain fatty acids (SCFAs), as well as restoring redox homeostasis by activating antioxidant enzymes (SOD, CAT, GPx).

Thirdly, it should be emphasized that *L. acidophilus* is increasingly used as a biological production device enabling the green synthesis of nanoparticles (e.g., silver, selenium), which are characterized by high selective cytotoxic activity (e.g., against Caco-2, A549, HepG2 lines). Furthermore, the synergy of *L. acidophilus* with thiolated chitosan carriers conjugated with folic acid (in the form of N/5FU/Se@FTCsNPs) allows for the targeted release of chemotherapeutic drugs (e.g., cytostatics such as 5-Fluorouracil) directly to the target site (tumors or tumor microenvironment), which significantly reduces the overall toxicity of chemotherapy and improves the quality of life of patients by strengthening the integrity of the intestinal barrier (increasing the production of Mucin 2 and tight junction proteins). Epithelial barrier integrity is another common element of antiallergic protection and oncological prevention. By increasing the expression of barrier proteins and mucins (e.g., Mucin 2), *L. acidophilus* may prevent the translocation of endotoxins that contribute to the development of liver cancer (via the gut–liver axis). It may also inhibit the penetration of allergens into the bloodstream, thereby reducing excessive sensitization of the immune system.

Despite the extremely promising research results, it should be emphasized that most of the cited scientific evidence is based on *in vitro* cellular models and *in vivo* animal models.

To enable widespread and safe use of *L. acidophilus* in the treatment of allergic diseases and cancer, it is crucial to conduct multicenter, randomized clinical trials (RCTs) in large and diverse patient groups, which will ultimately confirm the promising preclinical results.

Proceeding to the clinical phase requires also a thorough understanding of the interaction of *L. acidophilus* with conventional anticancer drugs, such as selective estrogen receptor modulators (e.g., tamoxifen), which have been shown to reconfigure the mammary gland tissue microbiome (*glandula mammaria*), exploiting the presence of probiotic bacteria to inhibit proliferation (Ki67). Future research should also focus on precisely mapping the strain specificity of *L. acidophilus*, which will allow us to determine which specific bacterial strains demonstrate the highest efficacy in specific disease entities and how these strains react in synergy with classical pharmacotherapy (e.g., as adjuvants in allergy immunotherapy or cancer chemotherapy).

In summary, *L. acidophilus* may serve as a valuable component of probiotic-based oncological therapy. When administered in appropriate doses, it may confer specific health benefits, including reducing cancer risk, promoting remission, and inhibiting metastasis. The integration of *L. acidophilus* with nanotechnology, along with its ability to improve the homeostasis of the intestinal microbiota and directly suppress oncogenes (e.g., mTOR, VEGF), makes this probiotic one of the most versatile and safe components of modern medicine, with potential future applications in the treatment of cancer and chronic inflammatory and allergic diseases.

## Figures and Tables

**Table 1:** The mechanism of action of *L. acidophilus* and its effects on the modulation of cellular and humoral immunity.

*Lactobacillus acidophilus* Strain	Type of Research	Research Model	Mechanism of Action → Obtained Result	References
**LA-5**	*in vitro*	dendritic cells (DCs)	Stimulation of lipopolysaccharide-activated dendritic cells → downregulation of gene transcription (including *BIRC3*, *CASP1*, *NLRP3*, *RIPK1*) → reduction in inflammatory response and apoptosis.	[21]
			Stimulation of non-lipopolysaccharide-activated dendritic cells to produce cytokines IL-1β, IL-6, IL-12, and CXCL8 → induction of the “immune priming” effect—stimulating macrophages and activating T lymphocytes for a defensive response.	
**PTCC CS/F/721/01/01**	*in vitro*	monocytes from women with endometriosis	Reduction in pro-inflammatory IL-1 and IL-6 levels in monocytes of women with endometriosis → downregulation of the inflammatory response.	[22]
**n/a**	*in vitro*/*in vivo*	ovariectomized female BALB/c mice	Increase in pTreg cells and decrease in tTreg cells in the lamina propria of the small and large intestines, mesenteric lymph nodes, and bone marrow → reduction in inflammation and attenuation of osteoclast activity.	[24]
**n/a**	*in vitro*/*in vivo*	pigs ((Landrace × Yorkshire) × Duroc)	Reduction in the number of CD4+ (helper) and CD8+ (cytotoxic) T lymphocytes → reduction in immune system hyperreactivity.	[26]
			Suppression of the TLR4/NF-κB pathway in peripheral blood mononuclear cells (PBMCs) → reduction in TNF-α and IL-6 production → attenuation of inflammation.	
			Downregulation of IFN-γ, IL-8, and IL-1β1 cytokine expression (upon lipopolysaccharide exposure) and upregulation of IL-4 and IL-10 cytokines → attenuation of inflammation.	
**FCQHC4L1**	*in vitro*/*in vivo*	male C57BL/6 mice	Increased expression of the Muc2 protein → strengthening of intestinal mucosal homeostasis.	[27]
			Inhibition of GRP78/ATF6 and GRP78/IRE1/XBP1 pathway activity → improvement of intestinal barrier tightness.	
			Stimulation of intestinal microbiota to secrete butyric acid → provision of energy for enterocytes and colonocytes.	
			Reduction in IL-6 and TNF-α levels and increase in IL-10 levels in the large intestine → reduction in inflammation.	
**LA1**	*in vitro*/*in vivo*	caco-2 cells; Wild-type mice	Inhibition of NF-κB p50/p65 heterodimer gene activation → neutralization of the pro-inflammatory effects of TNF-α.	[28]
			Inhibition of MLCK (myosin light chain kinase) expression → prevention of actin-myosin ring contraction within the cell → preservation of tightly formed tight junctions (TJs) → increased intestinal barrier tightness.	
			Activation of the PI3K (phosphatidylinositol-3-kinase) pathway and IKK-α phosphorylation → stimulation of the TLR-2 receptor to block the pro-inflammatory action of TNF-α.	
			Activation of NF-κB p50/p65 (dependent on TLR-2 and MyD88 protein) in immune cells → recognition of threats and defense against infection.	
**ATCC 4356**	*in vitro*/*in vivo*	BALB/c male mice	Increased MUC-1 levels in gastric epithelial cells → protection of the epithelium against damage and pathogen interaction.	[29]
			Reduction in excessive GAL-3 (galectin-3) levels → reduction in the risk of tumor development.	
**Multi-strain probiotics containing *L. acidophilus***	*in vitro*/*in vivo*	calves and piglets	Increased number of B lymphocytes in lymph nodes, spleen, tonsils, bone marrow, and peripheral blood → increased humoral production of IgA and IgG.	[13]

**Table 2:** Beneficial effects of probiotics reported in preclinical studies.

Probiotic (Strains)	Associated Health Benefits	Mechanism of Action → Obtained Result	Experimental Model	References
***Lactobacillus acidophilus* (NCFM)**	Alleviation of food allergies and inflammatory bowel conditions.	Suppression of Th2-type response → reduction in IgE and histamine levels; Induction of regulatory T cells (Tregs) → expression of anti-inflammatory cytokines (e.g., TGF-β1).	*In vivo*: Female BALB/c mice	[31]
***Lactobacillus acidophilus* (KLDS 1.0738)**	Attenuation of food hypersensitivity in the treatment of cow’s milk allergy (CMA).	Induction of miR-146a overexpression → inhibition of the pro-inflammatory TLR4/NF-κB signaling pathway → reduction in the production of inflammatory cytokines.	*In vitro*: Macrophages; *In vivo*: Female BALB/c mice (6–8 weeks old)	[32]
***Lactobacillus acidophilus* (CGMCC 0460.2)**	Suppression of food allergen hypersensitivity.	Inhibition of Th2-type immune response → inhibition of IL-4 and IL-10 cytokine production by Th2 helper T cells.	*In vivo*: Pregnant Sprague Dawley rats	[33]
***Lactobacillus acidophilus* (CICC 6081) + *Lactobacillus plantarum* subsp. *plantarum* (CICC 20988)**	Inhibition of excessive immune response to food allergens.	Restriction of Ca^2+^ ion influx from the extracellular space and endoplasmic reticulum into effector cells (mast cells and basophils) → inhibition of the release of histamine, tryptase, platelet-activating factor, prostaglandins, and leukotrienes → inhibition of allergic symptoms.	*In vitro*: Isolated human basophilic leukemia cells (KU812 cell line)	[39]
***L. acidophilus* (ATCC 4356)**	Anti-inflammatory activity in gastric epithelial cells.	Increased expression of interleukin 17 (IL-17) → inhibition of the transformation of healthy cells into neoplastic cells.	*In vivo*: 24 BALB/c male mice; *In vitro* and *in vivo*	[29]
***Lactobacillus acidophilus* (LA1)**	Supportive treatment of inflammatory bowel disease (IBD).	Reduction in pro-inflammatory cytokine TNF-α activity → protection of intestinal barrier integrity.	*In vitro*: Caco-2 cells	[28]
***L. acidophilus* (ATCC 4356)**	Limitation of colorectal cancer (CRC) development and metastatic risk.	Increased expression of *BAX*, *CASP3*, and *CASP9* genes → initiation of programmed cell death (apoptosis) in cancer cells; Reduction in Bcl-2 titers → release of cytochrome c from mitochondria and activation of the caspase cascade → apoptosis. Reduction in MMP-2 and MMP-9 expression → inhibition of cancer cell metastasis; Increased expression of miR-34 and let-7 → limitation of tumor growth; Increased suppression of miR-21 and miR-155 → inhibition of tumor growth and metastasis.	*In vitro*: HT-29 (Human Colorectal Adenocarcinoma) *In vitro*: SW480 (Human Colorectal Adenocarcinoma)	[46]
**Exopolysaccharide LA-EPS-20079 from *L. acidophilus* DSMZ 20079**	Prevention and therapy of colorectal cancer (CRC).	Downregulation of *BCL2* and *Survivin* gene expression → apoptosis of neoplastic cells.	*In vitro*: Caco-2 colon cancer cell line	[50]
**Exopolysaccharides produced by *L. acidophilus***	Prevention and therapy of colorectal cancer (CRC).	Increased activity of SOD, CAT, and GPx; regeneration of vitamin C and GSH levels → restoration of redox homeostasis → inhibition of oncogenic processes → reduction in carcinogen-induced neoplastic polyp formation.	*In vivo*: Male Sprague–Dawley rats	[51]
***L. acidophilus* (CUL 60) + Pterostilbene**	Colorectal cancer prevention.	Inhibition of the development of early neoplastic lesions (aberrant crypt foci) caused by carcinogens.	*In vivo*: Male Wistar rats (*Rattus norvegicus albinus*)	[52]
***L. acidophilus* + Calcium citrate + *Moringa oleifera* leaf extract**	Inhibition of intestinal carcinogenesis and prevention of carcinogen-induced hepatotoxicity.	Production of short-chain fatty acids (SCFAs) by the probiotic → limitation of inflammatory processes; Inhibition of *CTNNB1* gene expression and β-catenin signaling pathway → G1 phase cell cycle arrest→ prevention of S-phase entry (DNA replication and division). Calcium ion-induced overexpression of CaSR → increased E-cadherin expression and inhibition of the Wnt/β-catenin pathway → inhibition of CRC cell proliferation. Induction of thioredoxin-interacting protein (TXNIP) by D-allose (from *M. oleifera*) → stabilization of p27kip1 protein → inhibition of neoplastic cell growth (G1 phase).	*In vivo*: Sprague Dawley rats; *In vitro*: Colo 205 (human colorectal cell line)	[54]
***L. acidophilus* KCTC 3171 + *B. bifidum* KCTC 3202 + Pistachio milk + Inulin**	Colorectal cancer prevention.	Degradation of fructans (inulin) to acetate → overexpression of caspase-3 → reduction in α-tubulin levels → destabilization of the cytoskeleton → apoptosis of cancer cells.	*In vitro*: Caco-2 cells	[59]
***L. acidophilus* ATCC 4356 + *B. bifidum* ATCC 29521 + Nisin + 5-Fluorouracil**	Therapy for colorectal cancer (CRC).	Induction of reactive oxygen species (ROS) overproduction → severe oxidative stress → apoptosis of neoplastic cells; Increased *PTEN* gene expression → inhibition of the PI3K/AKT/mTOR pathway → inhibition of neoplastic cell growth and division; Increased *CASP9* gene expression → caspase 9 synthesis→ initiation of apoptosis; Increased suppression of mTOR and VEGF-α pathways→ attenuation of angiogenesis and tumor proliferation.	*In vitro*: CT26 and L929 cells; *In vivo*: Male BALB/c mice	[60]
***Lactobacillus acidophilus***	Regulation of metabolic homeostasis in colorectal cancer models.	Reduction in TG and LDL in the blood → limitation of lipid availability → inhibition of neoplastic cell proliferation; Inhibition of *LPR* gene expression → decreased SIRT1 expression → apoptosis; Upregulation of the *VDR* (Vitamin D Receptor) gene → increased calcitriol binding and activity → inhibition of the Wnt/β-catenin pathway → restriction of neoplastic cell proliferation.	*In vivo*: Male BALB/c mice	[55]
***L. acidophilus* CGMCC 878 (L.A 878)**	Inhibition of carcinogenic toxin activity in the intestines; CRC prevention and therapy.	Modulation of microbiota: reduction in *Ruminococcus obeum*, *Clostridium thermocellum*, *Bacteroides vulgates*, etc., and increase in *Lactobacillus reuteri* → attenuation of colorectal tumor development; Downregulation of β-glucuronidase expression → reduced metabolism of carcinogens → limitation of intestinal damage.	*In vivo*: male Sprague Dawley rats	[49]
***Lactobacillus acidophilus* (ATCC 43)**	Supportive treatment for ulcerative colitis; Reduction in CRC risk.	Reduction in TNF-α, IL-1β, IL-6, and IFN-γ levels → attenuation of inflammation; Increased IL-10 levels → reducing inflammation; Downregulation of miR-1, miR-let-7d, and miR-99a → inhibition of cell division and migration → limitation of metastasis; Downregulation of miR-155 → silencing of intestinal inflammation.	*In vivo*: Wistar rats	[65]
**Exopolysaccharides from *L. acidophilus* (ATCC 4356)**	Inhibition of hepatocellular carcinoma (HCC) development.	Restriction of TLR-2 activity, STAT3 phosphorylation, and p38 MAPK activity→ attenuation of inflammation → limitation of neoplastic cell proliferation; Reduction in IL-17 and TGF-β1 levels → inhibition of liver fibrosis and neoplastic changes.	*In vivo*: Male rats	[61]
***Lactobacillus acidophilus* (#DSM 20079)**	Inhibition of hepatocellular carcinoma (HCC) progression.	Production of valeric acid by LA → activation of hepatocyte GPR41 and GPR43 receptors → inhibition of the Rho-GTPase pathway → activation of the p38 kinase pathway → inhibition of neoplastic cell survival and migration.	*In vivo*: Male C57BL/6 mice	[62]
***Lactobacillus acidophilus* (CICC 20244)**	Enhancement of virotherapy in HCC treatment.	Increased number of dendritic cells and CD8+ T lymphocytes → enhanced production of perforin, granzyme B, IFN-γ, and TNF-α → induction of apoptosis in neoplastic cells.	*In vivo*: Female C57BL/6J mice	[63]
***L. acidophilus* LA-5 + Organic propolis extract**	Prevention and therapy for breast cancer.	Proliferation of T and B lymphocytes, macrophages, and dendritic cells in the spleen → production of specific antibodies → apoptosis of neoplastic cells → reduction in tumor size. Increased IFN-γ secretion → enhanced synthesis of IL-2, IL-6, and TNF-α → apoptosis → reduction in breast tumor size.	*In vitro*: 4T1 (murine breast cancer cells); *In vivo*: Balb/c mice	[70]
***L. acidophilus* ATCC 4356 + *L. casei* ATCC 39392 + Vitamin D3**	Increasing the therapeutic index of cytostatic drugs.	Upregulation of Bax and caspase 3 activity; suppression of the *Bcl-2* gene → reduction in breast tumor mass and volume.	*In vivo*: Female BALB/c inbred mice; *In vitro*: 4T1 mouse carcinoma cell line	[71]
***Lactobacillus acidophilus***	Neutralization of mycotoxins (e.g., aflatoxin M1) contaminating breast milk.	Removal of carcinogens from breast milk → attenuation of mutagenic and carcinogenic processes.	*In vitro*: Human breast milk samples	[72]
***Lactobacillus* and *Bifidobacterium* species**	Inhibition of mammary gland carcinogenesis.	Downregulation of Ki-67 protein expression → decreased breast cell proliferation → reduction in tumor formation.	*In vivo*: Female BALB/c and B6.MMTV-PyMT mice; *In vitro*: MCF-7 and ZR-75-1 cell lines	[73]
***L. acidophilus* (IIA-2B4) (isolated from raw beef)**	Prevention and therapy for cervical cancer.	Induction of deformation and disintegration of neoplastic cells.	*In vitro*: HeLa cells	[67]
***L. acidophilus* culture supernatant**	Prevention and therapy for cervical cancer.	Upregulation of *BAX* and downregulation of *BCL2* expression → increased caspase-3 expression → proteolysis → apoptosis; Downregulation of *MMP9* expression → limitation of metastasis.	*In vitro*: CaSki cell line	[66]
***L. acidophilus* LA-5 + *B. animalis* subsp. *lactis* BB-12**	Prevention of acute radiation-induced diarrhea (RID) in cervical cancer patients.	Reduction in radiation-induced intestinal epithelial cell apoptosis and enhancement of innate immune response in the gut → protection against pathogen colonization; Stimulation of lactase production → support for lactose digestion.	Study population (*n* = 74)	[68]
**Cell-free supernatant from *L. acidophilus* (LACFS)**	Cervical cancer prevention.	Induction of cell shrinkage, membrane blebbing, and loss of adhesion to the substrate → neoplastic cell death.	*In vitro*: SiHa cell line	[69]
***L. acidophilus***	Therapy for squamous cell carcinoma (SCC).	Activation of the TRAIL cytokine → apoptosis of oral squamous cell carcinoma cells.	*In vitro*: Human HNSCC cells of the oral cavity (HNO97 cell line)	[76]
***L. acidophilus*/Cell-free supernatant**	Antigenotoxic effects in the prevention and treatment of Barrett’s esophagus (esophageal adenocarcinoma).	Inhibition of NF-κB activity → attenuation of inflammatory response → reduction in DNA damage → downregulation of carcinogenesis in esophageal tissues.	*In vivo*: Mice	[77]
***Lactobacillus acidophilus* (La-14 SD-5212)**	Anti-tumor activity against gastric tumors.	Reduction in COX-2 expression → inhibition of tumor angiogenesis → apoptosis.	*In vitro*: Gastric adenocarcinoma cell line (AGS)	[78]
***Lactobacillus acidophilus* (AJ2)**	Prevention of bone resorption loss by osteolytic tumors.	Increased IFN-γ secretion from immune cells → restriction of bone tumor growth and metastasis.	*In vivo*: Humanized BLT (Bone Marrow-Liver-Thymus) mice; *In vitro*: MiaPaCa-2 (MP2) tumor cells	[14]
**Tyndallized *L. acidophilus***	Protective effect against skin cell photoaging.	Suppression of MMP-1 and MMP-9; inhibition of the MAPK pathway → inhibition of neoplastic cell proliferation and migration; reduction in transepidermal water loss and stimulation of collagen synthesis.	*In vivo*: HR-1 male mice	[79]
***Lactobacillus acidophilus* (L-92)**	Inhibition of allergen-induced passive (PCA) and active (ACA) cutaneous anaphylaxis.	Suppression of IgE titers → inhibition of cutaneous anaphylaxis; Reduction in mast cell and eosinophil infiltration → attenuation of inflammation in the mucosa and dermal connective tissue; Restoration of cytokine homeostasis → downregulation of Th2 lymphocyte activity → inhibition of atopic dermatitis development.	*In vivo*: Albino ICR mice	[42]
***Lactobacillus acidophilus* (Moro) with “stealth” polymer coating**	Disruption of neoplastic cell homeostasis.	Reduction in L-lactate in tumor tissue → inhibition of the PI3K/AKT/mTOR pathway → energy deficit → apoptosis; Increased D-lactate secretion in M2 macrophages → polarization from M2 to M1 phenotype → phagocytosis of cancer cells.	In situ: Polymerization method; *In vitro*: 4T1 (Mouse Breast Carcinoma Cells)	[74]
**Silver nanoparticles (AgNPs) synthesized by *L. acidophilus***	Cytotoxic activity against colorectal, lung, and liver cancer.	Release of silver ions (Ag^+^) → increased production of reactive oxygen species (ROS) → damage to cellular proteins and lipids → apoptosis.	*In vitro*: Tumor cell lines: Caco, A549, and HepG2	[75]

## Data Availability

No new data were created or analyzed in this study.
